# Editing efficiencies with Cas9 orthologs, Cas12a endonucleases, and temperature in rice

**DOI:** 10.3389/fgeed.2023.1074641

**Published:** 2023-03-17

**Authors:** Eudald Illa-Berenguer, Peter R. LaFayette, Wayne A. Parrott

**Affiliations:** ^1^ Center for Applied Genetic Technologies, University of Georgia, Athens, GA, United States; ^2^ Department of Crop and Soil Sciences, University of Georgia, Athens, GA, United States; ^3^ Institute of Plant Breeding, Genetics and Genomics, University of Georgia, Athens, GA, United States

**Keywords:** genome editing, cas proteins, editing efficiency, specificity, temperature, heat treatment

## Abstract

The advent of CRISPR-Cas technology has made it the genome editing tool of choice in all kingdoms of life, including plants, which can have large, highly duplicated genomes. As a result, finding adequate target sequences that meet the specificities of a given Cas nuclease on any gene of interest remains challenging in many cases. To assess target site flexibility, we tested five different Cas9/Cas12a endonucleases (SpCas9, SaCas9, St1Cas9, Mb3Cas12a, and AsCas12a) in embryogenic rice calli from Taipei 309 at 37°C (optimal temperature for most Cas9/Cas12a proteins) and 27°C (optimal temperature for tissue culture) and measured their editing rates under regular tissue culture conditions using Illumina sequencing. StCas9 and AsCas12 were not functional as tested, regardless of the temperature used. SpCas9 was the most efficient endonuclease at either temperature, regardless of whether monoallelic or biallelic edits were considered. Mb3Cas12a at 37°C was the next most efficient endonuclease. Monoallelic edits prevailed for both SaCas9 and Mb3Cas12a at 27°C, but biallelic edits prevailed at 37°C. Overall, the use of other Cas9 orthologs, the use of Cas12a endonucleases, and the optimal temperature can expand the range of targetable sequences.

## 1 Introduction

Modern plant breeding and agricultural biotechnology are experiencing a major revolution with the emergence of the clustered regularly interspaced short palindromic repeats (CRISPR)-associated (Cas) endonucleases. This technology enables modifications of target DNA in a broad range of plant species. These include not only model plants, such *Arabidopsis*, *Brachypodium*, or *Medicago*, but also more than 70 crop species (Ricroch et al., 2017; Liu et al., 2021). The scope of CRISPR-Cas technology’s applications ranges from crop quality improvement (Ku and Ha, 2020; Liu et al., 2021) to abiotic (Osakabe and Osakabe, 2017; Zafar et al., 2019) and biotic stress (Ahmad et al., 2021; Jain et al., 2021) management.

Despite the widespread adoption of gene-editing technologies, attaining high editing efficiency on any gene of interest remains a challenge in many cases, particularly in large, complex, and partially duplicated plant genomes. For these, one of the major bottlenecks is finding adequate targetable sequences that meet the specificities of a given Cas nuclease.

Successful and specific targeting relies on a unique protospacer adjacent motif (PAM) adjacent to each target sequence. The Cas9 from *Streptococcus pyogenes* (SpCas9), the most commonly used Cas, requires a short 5′-NGG-3′ PAM sequence for targeting (Jinek et al., 2012). The simplicity of this PAM sequence increases the editing feasibility of the target DNA but leads to unintended edits if there are multiple copies of the target sequence, as happens in complex genomes.

Thus, the characterization of Cas9 orthologs, the use of Cas12a nucleases, and the application of rational protein engineering have expanded the range of targetable sequences. To date, almost 800 different Cas9 (Gasiunas et al., 2020; Makarova et al., 2020) and 58 distinct Cas12a orthologous (Zetsche et al., 2015; Marshall et al., 2018; Teng et al., 2019; Jacobsen et al., 2020; Zetsche et al., 2020) have been identified. In addition, 59 Cas effectors have been engineered to recognize a more flexible PAM (Collias and Beisel, 2021).

Much effort has been dedicated to increasing the frequency of induced mutations at the target site, including temperature optimization (Xiang et al., 2017; Milner et al., 2020). Cas9 and Cas12a enzymes are sensitive to temperature (LeBlanc et al., 2018; Malzahn et al., 2019), and changes in temperature affect the on-target mutation rate and increase off-target editing rates (Xiang et al., 2017; LeBlanc et al., 2018; Banakar et al., 2022; Blomme et al., 2022). Their optimal temperature coincides with that of their mammalian commensals, which is at least 10°C too high for most plant tissue cultures. With these precedents in mind, we aimed to assess which Cas9/Cas12a proteins and incubation temperature are best for editing calli derived from rice.

## 2 Materials and methods

### 2.1 Plant material and growing conditions

Rice seeds (*Oryza sativa* L. cv Taipei 309) were used to start cell lines as described by Phan et al. (2007). Seeds were dehusked and surface-sterilized in 70% (v/v) ethanol for 2 min followed by a 30-min soak incubation in 60% household bleach (6.0% sodium hypochlorite) with 0.01% Tween-20. After rinsing three times with sterile H_2_O for 2 minutes, the kernels were dried and plated in a 5 × 5 grid on modified NB medium (Chen et al., 1998) solidified with 2.5 g L^-1^ Gelzan™ (BioWORLD, Dublin, OH, USA) in 100 × 15 mm Petri dishes sealed with 3M™ Micropore™ tape (3M Healthcare, Saint Paul, MN, US). Plates were incubated at 27°C in dark conditions to induce callus formation. Type II calli (Armstrong and Green, 1985) were selected and maintained in the dark with transfers every 3 weeks for 5 months before transformation.

### 2.2 Vectors

**FIGURE 1:**
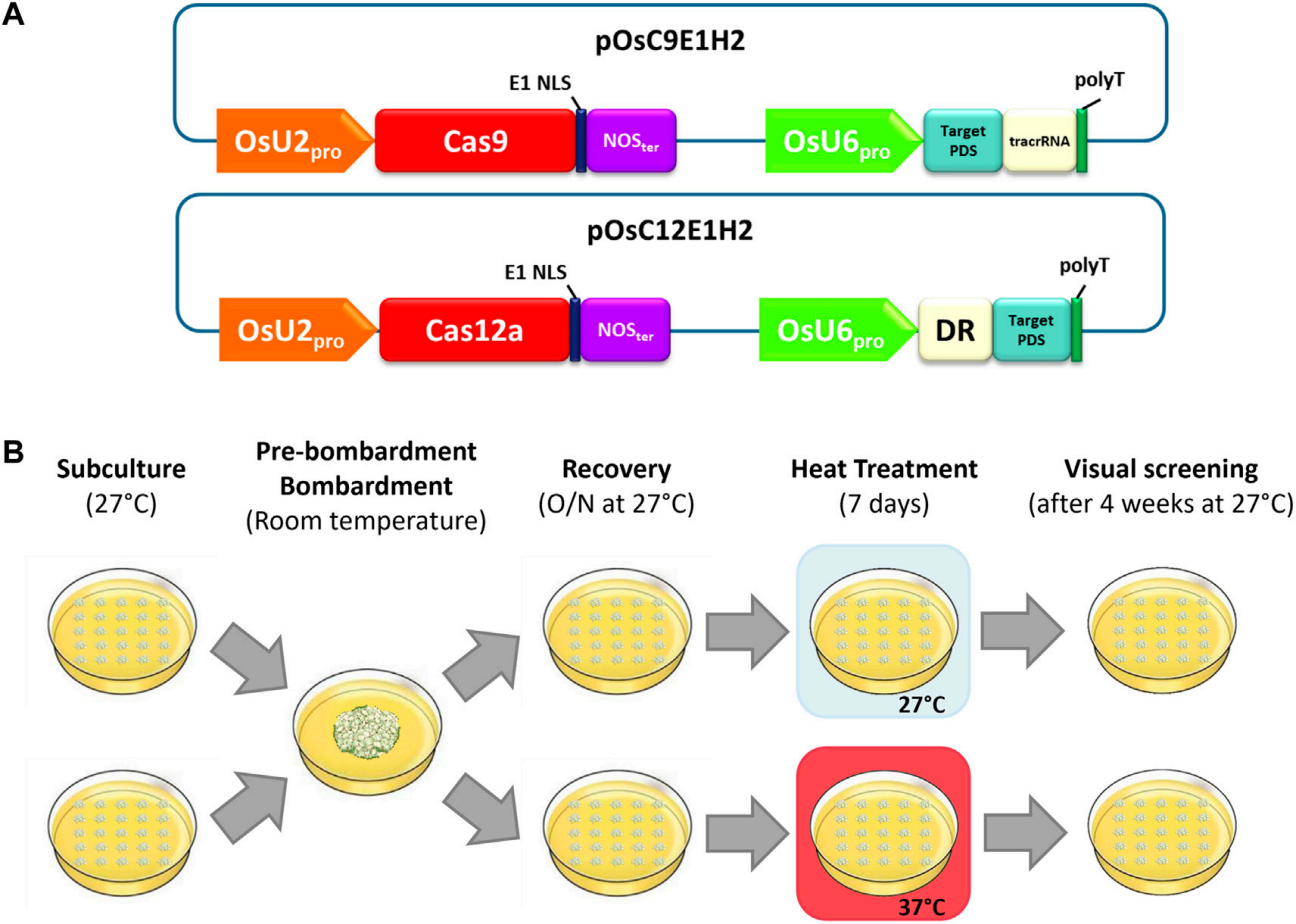
Experimental design overview. **(A)** Schematic representation of the plasmids used for stable biolistic transformation. **(B)** Workflow of temperature treatment for assessing Cas9/Cas12a editing rates.

The editing vectors (Figure 1A) were modified versions of pOsC9-H2, which contain the OsUbi2 promoter:Cas9 (*Sbf* I/*Xba* I fragment from pRGEB32 Addgene plasmid #63142, Xie et al. (2015)) and the PCR-amplified nopaline synthase (NOS) terminator inserted into a modified pMECA vector (Thomson and Parrott, 1998). The PvUbi2 promoter:*hph* (*Sbf* I/*Xho* I fragment from pPANIC10A (Mann et al., 2012) and the PCR-amplified Pv*Ub*i*2* terminator (GenBank HM209468) were then added to complete the plant selectable marker cassette.

Vector pOsSpC9E1-H2 was assembled by inserting a T4 DNA polymerase-blunted *Bam* HI/*Avr* II fragment of pStuHSpyCas9 (Campbell et al., 2019) containing SpCas9 and the E1 NLS from Glyma.06g207800 to replace the Cas9:NLS in pOsC9-H2 digested with *Bsr* GI/*Not* I and blunted with T4 DNA polymerase.

Next, pOsSaC9E1-H2 was assembled as above from pStuHSaC9 in which *Staphylococcus aureous* Cas9 (SaCas9, Addgene plasmid #61591) replaced SpCas9 in pStuHSpyC9. Plasmid OsSt1C9E1-H2 contains the *Streptococcus thermophilus* #1 Cas9 (St1Cas9, Addgene plasmid #48669), pOsAsC12aE1-H2 contains the *Acidaminococcus* AsCas12a (Addgene plasmid #69982) and pOsMb3C12aE1-H2 contains the *Moraxella bovoculi* AAX11_00205 Mb3Cas12a (Addgene plasmid #92293).

The gRNA cassette containing the OsU6 promoter (Feng et al., 2013), the specific target site sequences, and the corresponding trans-activating crRNA (tracrRNA) scaffold or direct repeat (DR) sequence for each endonuclease (Supplementary Table S1) was inserted into an *Asc*I/*Pme*I-digested modified pOsC9-H2 plasmid *via* Gibson assembly as described in Campbell et al. (2019).

PCR for cloning used Q5 Polymerase (New England Biolabs, Ipswich, MA, United States) and was verified with Sanger sequencing (Genewiz, Inc.). NEB 5-α competent *E. coli* (New England Biolabs, Ipswich, MA, United States) was used as host for cloning and plasmid propagation. Bacteria were grown in Luria-Bertani (*LB*) medium supplemented with 100 μg mL^-1^ ampicillin.

#### 2.2.1 Target selection for protein activity assessment

Phytoene desaturase (PDS) from rice (OsPDS, LOC_Os03g08570, Os03g0184000) was selected as the target gene to evaluate Cas nuclease activity at different temperatures. OsPDS is encoded by a single copy gene that consists of 14 exons and spans 4,416 bp on rice chromosome 3. The PDS gene is commonly targeted because its disruption produces a bleached phenotype due to the absence of chlorophyll-protecting carotene. The bleached color serves as a marker to visually score CRISPR-Cas activity.

One single target was selected for each Cas ortholog (except AsCas12a and Mb3Cas12a, which share the same target site, Table 1). An appropriate target site was selected from the sites that displayed the lowest predicted off-target rate and the highest predicted on-target activity. gRNA expression through OsU6 promoter requires a guanosine nucleotide to initiate transcription. Geneious 11.0.5 (https://www.geneious.com) was used to identify all possible candidate targets for each Cas protein [GN19-NGG (SpCas9), GN20-NNGRRT (SaCas9), GN19-NNAGAAW (St1Cas9), TTTV-N20 (As/Mb3Cas12a)] and their potential off-targets, with a maximum mismatch tolerance against off-targets of six or fewer nucleotides and up to 1 indel. *O. sativa* Japonica Group (Japanese rice) genome assembly IRGSP-1.0 from The International Rice Genome Sequencing Project (IRGSP) (Kawahara et al., 2013) was used for off-target site prediction. Potential on-target activity was determined using Azimuth 2.0 (Doench et al., 2016; Sanson et al., 2018) for SpCas9 and SaCas9, CCTop (Stemmer et al., 2015; Labuhn et al., 2017) for St1Cas9 and Seq-DeepCpf1 (Kim et al., 2018) for both Cas12a endonucleases.

**TABLE 1:** Molecular analyses of OsPDS alleles in rice embryogenic calli exposed to heat treatment.

Cas effector	Temperature (°C)	Initial explants	Hyg^R^ callus	Hyg^R^ events	Non-analyzed events	Events with no reads	Events analyzed	Edited events	Edition			
									Monoallelic	Biallelic		Multiple alleles
										HOM	HET	
SaCas9	27	125	22	33	0	1	30	8	4	1	0	3
	37	125	36	80	2	1	77	26	11	8	4	3
SpCas9	27	125	50	121	5	0	116	73	8	19	33	13
	37	125	60	140	1	0	139	78	11	12	38	17
St1Cas9 **(Esvelt)**	27	125	29	79	1	1	77	0	0	0	0	0
	37	125	41	118	1	1	116	0	0	0	0	0
St1Cas9 **(Briner)**	27	125	24	55	2	0	53	0	0	0	0	0
	37	125	22	44	1	0	43	0	0	0	0	0
AsCas12a	27	125	40	86	2	0	66	1	0	0	0	1
	37	125	48	96	1	0	95	2	1	0	1	0
Mb3Cas12a	27	125	45	96	1	1	94	5	4	1	2	0
	37	125	52	130	2	1	127	55	9	5	27	14

Abbreviations: Hyg^R^ stands for hygromycin-resistant tissue, HOM denotes homozygous biallelic edits, and HET indicates heterozygous biallelic edits.

### 2.3 Biolistic transformation

Type II rice callus was transformed using biolistic microprojectile bombardment 4 days after the last subculture. Prior to transformation, for each construct, 50 rice calli were transferred onto NB osmotic medium (NBO, Chen et al., 1998) and placed for 6 h in a 2-cm diameter disk in the center of a Petri dish.

Transformation was achieved using the Biolistic PDS-1000/He particle delivery system (BIO-RAD, Hercules, CA) at a target distance of 9 cm and helium pressure of 7,584.233 kPa (1,100 psi). For each shot, 0.3175 mg of 0.4-µm gold particles (InBio Gold, Victoria, Australia) and 125 ng of plasmid DNA were used. Briefly, 5 mg of gold particles were washed with 1 mL of 100% ethanol. The microcarriers were suspended by water bath sonication for 10 s and placed on ice for 30 s. The sonication and ice step was repeated a total of three times. The gold particles were recovered by centrifugation at 3,287 ×g for 5 min and resuspended in 175 µL of 100% ethanol. Two rounds of vortexing for 1 min and water bath sonication for 10 s were conducted prior to withdrawing a 40 µL aliquot to a new sterile microfuge tube. A short centrifugation was carried out to settle the microcarriers, and the supernatant was removed. The gold particles were washed in 1 mL sterile water followed by a 5-min centrifugation step at 268 ×g to recover the gold prep. After spinning, 400 ng of plasmid DNA, 220 µL sterile water, 250 µL of 2.5 M CaCl_2_, and 50 µL of 100 mM spermidine were added to the microcarriers. The gold suspension was homogenized after the addition of each solution by vortexing for 2 s and sonicating for 10 s. DNA/gold suspension was precipitated on ice for 2 min, pelleted by centrifugation at 43 ×g for 5 min, washed in 600 µL 100% ethanol at 43 ×g for 5 min, and finally resuspended in 36 µL 100% ethanol. The microcarriers were incubated on ice for 1 h. Ten µL of DNA-coated gold beads were spread on a macrocarrier and allowed to dry.

The six different plasmids were shot the same day using a randomized block design, with five replicates. Each treatment consisted of one Cas endonuclease whose activity was assessed at two different temperatures (27°C and 37°C). The experimental unit consisted of a 100 × 15 mm Petri dish containing 25 type II embryogenic callus pieces.

Eighteen hours post-bombardment, tissue from each shot was transferred from NBO medium onto two 100 × 15 mm Petri dish plates of modified NB medium containing 50 mg/L hygromycin B (Calbiochem^®^, NBH50) in a 5x5-grid. Plates were divided into two groups: one group was subjected to heat treatment (37°C for 1 week) and then moved to 27°C, while the other group was kept at 27°C. In both cases plates were maintained in the dark. PDS *knockout* phenotypes were discernable by their white color and were visually scored 4 weeks after moving plates to 27°C (Figure 1B).

### 2.4 Tissue culture genotyping

Genomic DNA from all hygromycin-resistant calli was isolated using the CTAB method adapted from Stewart and Via (1993). In summary, callus pieces were ground with 900 µL CTAB buffer (100 mM Tris-Cl (pH 8.0), 20 mM EDTA, 1.42 M NaCl, 2% (w/v) cetrimonium bromide (CTAB), 2% (w/v) polyvinylpyrrolidone (PVP-40), and 4 nM diethyldithiocarbamicacid (DIECA)) with two 4.8-mm diameter steel beads (Med Supply Partners, Atlanta, GA, USA) in a GenoGrinder 2010 (Spex^®^ SamplePrep LLC, Metuchen, NJ, USA) for 1 min at 1,000 strokes min^-1^. The solution was incubated at 65°C for 30 min, after which 800 µL of chloroform:isoamyl alcohol (24:1) were added. The water-soluble fraction was recovered after centrifugation for 10 min at 16,000 ×g. DNA was precipitated with 0.85 volumes of 100% isopropanol and centrifugation as described before. Finally, the DNA pellet was washed twice with 70% ethanol, air-dried overnight, and resuspended in TE buffer. DNA quantity was measured with the BioTek™ Synergy™ 2 microplate reader (BioTek Instruments, Winooski, VT, United States).

A subset of 18 samples were analyzed by Sanger sequencing, using specific primers (Supplementary Table S2) to amplify the corresponding target regions in exon 5 and 10, respectively. The Synthego ICE Analysis tool v2 (Synthego, 2019) was used to detect the presence of CRISPR-Cas-induced mutations.

Editing patterns for all collected samples were then determined by deep sequencing of PCR amplification products using specific primers tailed with Illumina sequencing primers to check for both on-target and off-target cleavage (Supplementary Table S2) as described in Zhou et al. (2015). Indexed samples were pooled together and sequenced on the Illumina MiSeq platform (2x 150 cycles) at the Georgia Genomics and Bioinformatics Core (GGBC). Paired-end raw reads were pre-processed with Cutadapt v.3.4 (Martin (2011), with the settings -q 20 -m 125). Then, corresponding mates were merged with BBMerge function from the BBtools suite v.38.92 (https://jgi.doe.gov/data-and-tools/bbtools). Processed reads were analyzed using AGEseq software (Xue and Tsai, 2015). Statistical analyses were performed using R open-source software (version 4.2.0, R Core Team (2022)).

## 3 Results

**FIGURE 2:**
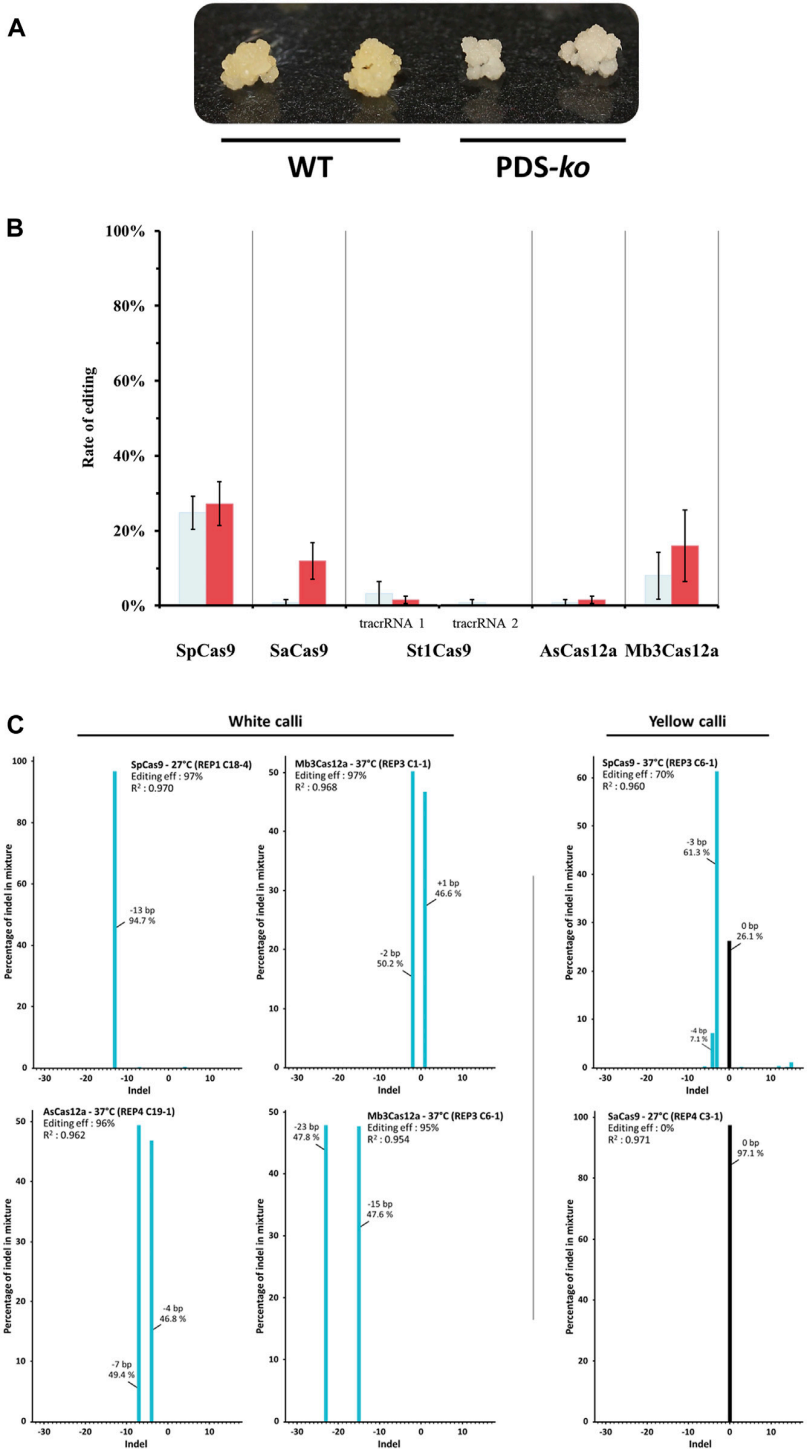
Gene editing using different Cas9/Cas12a variants. **(A)** Representative rice embryogenic callus phenotypes. Wild-type (WT) calli are yellow, while PDS-*ko* calli are white. **(B)** CRISPR-Cas gene editing efficiency of rice phytoene desaturase (OsPDS) gene both at 27°C (light blue) and 37°C (red). Bar graphs show the percentage of PDS mutants identified by transformed calli visual inspection. Standard error mean (SEM) is indicated by whiskers. St1Cas9 trans-activating CRISPR RNA (tracrRNA) reported by Esvelt et al. (2013) (tacrRNA 1) and Briner et al. (2014) (tacrRNA 2), respectively. **(C)** Indel distribution of insertions and deletions from single samples analyzed by Sanger sequencing.

Genome editing rates were assessed for five different RNA-guided endonucleases, SpCas9, SaCas9, St1Cas9, AsCas12a and Mb3Cas12a under two temperature regimes: 27°C and 37°C. The target gene for editing was the rice phytoene desaturase (OsPDS), as PDS knockouts display a detectable albino phenotype, allowing for quick screening based on tissue color (Figure 2A).

All possible 20-bp protospacer sequences targeting the OsPDS gene were identified for each Cas endonuclease, except SaCas9, which requires 21-bp protospacers (Supplementary Figure S1). Two hundred seventy-four targets were identified for Cas12a and 127 for SpCas9, both of which use shorter PAM sequences. Conversely, 31 and 7 target sequences were found for SaCas9 and St1Cas9, which recognize 6- and 7-nucleotide PAM sequences, respectively. Only a small subset of targets overlaps with coding sequences: 82, 48, 4 and 2 for Cas12a, SpCas9, SaCas9, and St1Cas9, respectively. To facilitate the direct comparisons between all Cas nucleases, we chose target sequences located within a 100-bp region on exon 5, on the premise that editing at any of these targets would have a similar impact on the protein structure and function. Predicted on-target activity and potential off-target sites were also considered, maximizing on-target activity and reducing off-target reactivity when choosing the target sites. Due to the reduced number of candidate protospacer sequences for St1Cas9, its the best target sequence is located on exon 10. Each target-specific crRNA was cloned into a biolistic vector with the rice ubiquitin 2 promoter driving the expression of the specific Cas nuclease, an E1 nuclear localization signal (NLS), and nopaline synthase (NOS) gene terminator (Figure 1A). Rice RNA polymerase III U6 promoter was used for single guide RNA (sgRNA) expression.

Constructs were shot into rice type II embryogenic calli. The experiment was conducted using a randomized complete block design with five replicates. There were 250 calli tested for each Cas endonuclease (125 per temperature treatment). One month after completing the different heat treatments, editing rates were assessed by visual scoring of calli to determine the number of calli that showed at least one edit present (i.e., 1 callus with 1 white event got the same score as 1 callus with 3 white events), and editing rates are summarized in Figure 2B.

SpCas9 is the best performing Cas enzyme, yielding the best editing rate, regardless of the incubation temperature (24.8% at 27°C and 27.2% at 37°C). Mb3Cas12a is the next best performing enzyme, albeit with relatively low efficiency at 27°C (8%) but reasonably high efficiency at 37°C (16%). The next best enzyme is SaCas9 at 37°C (12% efficiency), but its efficiency dropped to 0.8% at 27°C. St1Cas9 with the tracrRNA described by Esvelt et al. (2013) scored a 3.2% efficiency at 27°C, but only 1.6% efficiency at 37°C. The same protein using the alternative tracrRNA described by Briner et al. (2014) yielded <1% editing efficiency at 27°C and did not generate any edits at 37°C. Finally, AsCas12a produced a 1.6% editing rate at 37°C but only 0.8% at 27°C.

Tissue was collected for DNA extraction and molecular verification of editing from all newly formed hygromycin-resistant events (both white and yellow). In total, 1,072 samples were collected, including many events recovered from Cas transformations that showed low or no editing activity, as there could be underlying monoallelic edits that were missed in the visual scoring. Eighteen random events derived from each endonuclease were genotyped by Sanger sequencing. As expected, white tissue samples showed either homozygous or heterozygous biallelic mutations that knocked out PDS. Yellow tissue samples showed either no deletions (wt-like PDS) or, in some cases, editing in one of the alleles. These results prompted us to verify all available samples (Figure 2C).

**FIGURE 3:**
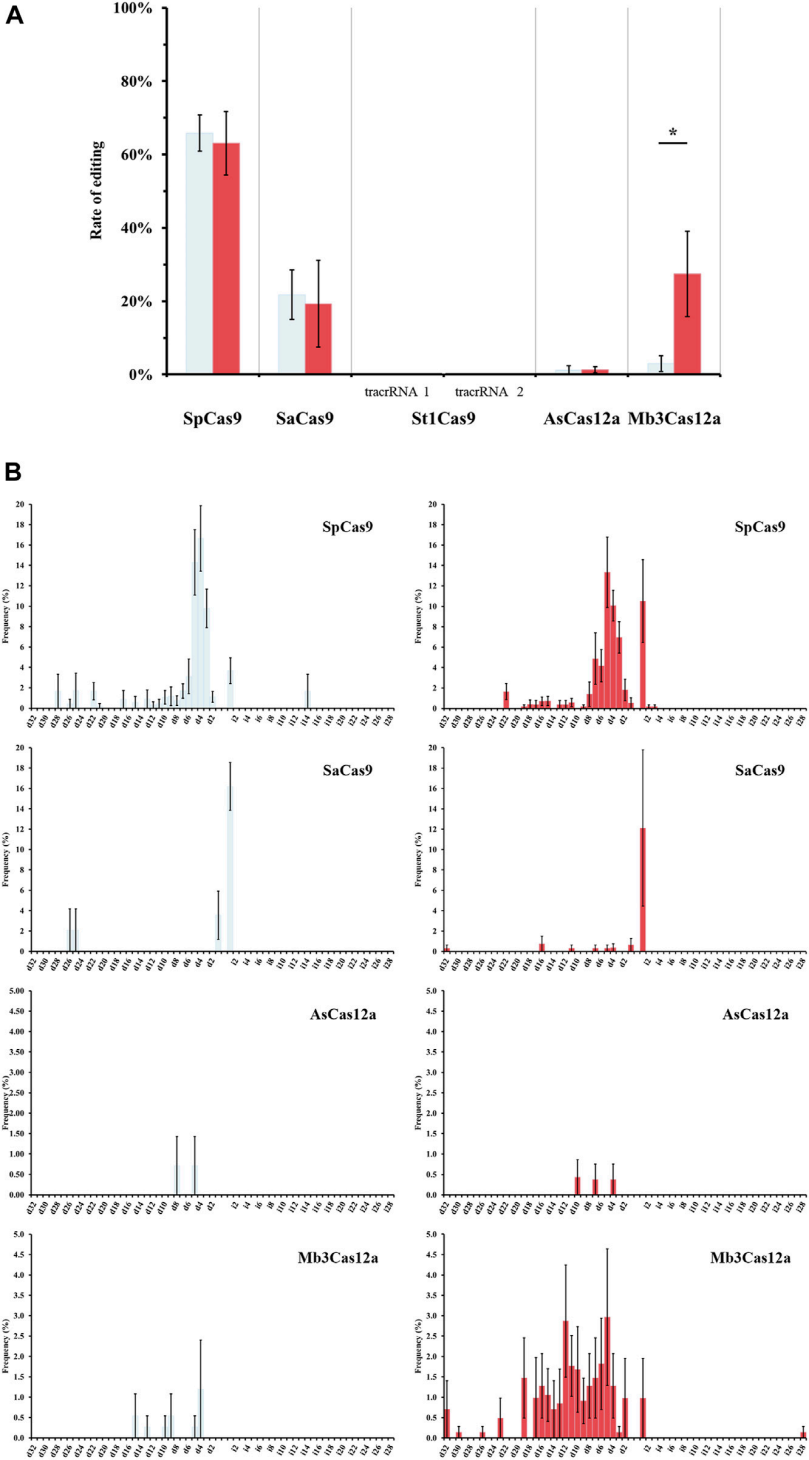
Gene editing efficiency of different Cas effectors *via* Illumina sequencing. **(A)** Editing frequencies of rice phytoene desaturase (OsPDS) gene under different temperatures, 27°C (light blue) and 37°C (red). Bar graphs show the percentage of PDS gene edits as identified by short-read sequencing. Trans-activating CRISPR RNA (tracrRNA) 1 and tracrRNA 2 were reported by Esvelt et al. (2013) and Briner et al. (2014), respectively. Significance *p*-value levels according to t-test are shown as <0.05 (*). **(B)** Indel size profiles. Occurrence of deletion (d) and insertion (i). Frequencies (as percentage shown in the *y*-axis) were calculated using the number of edited alleles with N bp deletions or insertions by the number of all the alleles identified by sequencing. Graphs depict means and error bars show standard error of the mean (SEM) from 5 replicates. Results from St1Cas9 are not shown due to the lack of editing.

Individual Illumina libraries were prepared for all the samples. Libraries were designed to include the target site, plus the three most likely off-target locations and were evaluated for each Cas protein. Illumina sequencing revealed some discrepancies from visual scoring (Figure 3A; Table 2, Supplementary Table S3), but all Sanger sequences were identical to their Illumina counterpart.

**TABLE 2:** Summary of the different target-specific crRNAs.

Endonuclease	PAM sequence	Spacer sequence (5' → 3')	Direction	Predicted on-target activity (Percentage)		Predicted off-target activity								
				CRISPRko^a^	CCTop^b^	Sequence (#1)	Off-target #1 Score	Location (#1)	Sequence (#2)	Off-target #2 Score	Location (#2)	Sequence (#3)	Off-target #3 Score	Location (#3)
SpCas9	CGG	GCGAGCTTGGTATTAATGAT	forward	54.47	56.24	TATATCTTGATATTAATGAT **AGG**	4.01	Chr02:28740598..28740620	GCGAGGTTGGTGATTAATGGT **GGG**	2.75	Chr10:19346533..19346556	GTAACCATGATATTAATGAT **AGG**	2.71	Chr01:35317259..35317281
SaCas9	CTGAAT	GTTTCAGGAAAATCAAACCGG	reverse	81.47	67.22	GTTAGAGTAAAATGAAACCGG **TTGAAT**	1.89	Chr11:22975874..22975900	GTTTCTGTTGGATCAAACCGG **TGGGAT**	1.62	Chr05:8034722..8034748	GATTGTGGTAGTTCAAACCGG**CTGGGT**	1.55	Chr03:27509680..27509706
St1Cas9	ATAGAAA	GTTTGAACATTTCAGGTTTG	forward	N/A	75.74	GCCTAAAGATTTTAGGTTTG **GCAGAAT**	1.73	Chr01:3663026..3663052	CTTTGAA-ATTAAAGGTTTG**ACAGAAT**	1.43	Chr08:25668255..25668280	GAACAAACAGTCCAGGTTTG **GAAGAAT**	1.36	Chr08:20390698..20390724
Cas12a^‡^	TTTA	AGTTGGAGCTTATCCCAACA	reverse	83.23	70.97	**TTTA**AGTTGGACCTTCTCCCAGCT	1.75	Chr03:6381960..6381983 (XP_015631410.1)	**TTTA**ATTTGGAGCTTAGCCATCCA	1.49	Chr05:4301398..4301421	**TTTC** AGCTGGAGCTCAGCCAAACA	1.38	Chr08:801967..801990

^a^ On-target scoring is performed using Azimuth 2.0 (Doench et al., 2016; Sanson et al., 2018) for SpCas9 and SaCas9 systems and Seq-DeepCpf1 (Kim et al., 2018) for Cas12a (https://portals.broadinstitute.org/gpp/public/analysis-tools/sgrna-design)

^b^ Stemmer et al. (2015); Labuhn et al. (2017) (https://crispr.cos.uni-heidelberg.de).

PAM sequences are highlighted in bold.

Underlined bases indicated mismatches between the spacer used and the putative off-target site.

^‡^ Same spacer was used for both AsCas12a and Mb3Cas12a.

When St1Cas9 was used with the tracrRNA described by Esvelt et al. (2013) a total of 197 newly formed hygromycin resistant events were recovered (118 events from heat-treated plus 79 events from tissue kept at 27°C). However, no evidence of editing was detected when the callus was sequenced, even though five transformed calli were initially identified as positively edited (presence of white callus colonies).

Similarly, when St1Cas9 was used with the tracrRNA described by Briner et al. (2014) no evidence of editing was detected among the 43 events recovered from tissue subjected to heat treatment or the 53 events recovered from tissue kept at 27°C. These findings agree with the nearly null activity observed visually. No edits were detected on any of the predicted off-target sites.

For SaCas9, the best observed editing rate (12%) occurred when the transformed tissue was incubated at 37°C for a week. The Illumina results show that this protein induces mainly 1-bp insertions (66%). Despite the prevalence of small mutations, monoallelic deletions up to 32-bp or biallelic deletions of 16-bp were also detected. Sequencing data confirmed what was visually observed, with the differences in editing activity detected between the two approaches being attributable to event heterozygosity that could only be detected by sequencing. However, the latter approach shows very similar SaCas9 activity levels at both tested temperatures (21.8% at 27°C and 19.3% at 37°C). No clear evidence of editing was observed for two of the three off-targets. Sequencing data retrieved for the third off-target was of extremely low quality and made it difficult to assess editing. However, the first off target from two replicates showed one consistent SNP located 7 bp downstream of the forward primer, not overlapping with the target sequence.

Finally, SpCas9 sequencing results confirm that activity levels are similar at both tested temperatures (65.9% at 27°C vs. 63.1% at 37°C). Editing rates are comparable to those obtained by visual scoring, with minor variation due to the presence of editing in heterozygosis and marginal error during tissue phenotyping. In contrast to SaCas9, SpCas9 primarly produces 3- to 5-bp deletions, although shorter and longer deletions were also observed. For instance, Sample C2-1 at 27°C from replicate 2 (PDS_Rep2_27C_Sp_C2-1) displays a biallelic edit: 22 bp and 26 bp deletions. Large insertions were also detected: sample C5-3 at 27°C from replicate 2 (PDS_Rep2_27C_Sp_C5-3) shows a monoallelic replacement of 14-bp that overlaps with the last section of the target. Interestingly, at 37°C we observed a significant increase in the number of 1-bp insertions (from 4% at 27°C to 11% at 37°C), rating close to 3- and 5-bp deletions (Figure 3B). Moreover, 30 events present multiple editing events, with up to six different alleles from editing. No strong evidence of editing at the first two off-target sites was observed, but, as described for SaCas9, samples from two replicates always have one SNP at the same position, in this case 6 bp downstream from the forward primer, with no overlap with the target sequence. As was also the case with SaCas9, reduced numbers of reads were obtained for the third off-target site, but in most cases, it was sufficient to confirm lack of editing. In 75 cases (29.4%), one or two reads presented SNPs localized on the target sequence, but since those are SNPs (as opposed to deletions) often located far from the predicted cut site of SpCas9 (between position 17–18 of the target sequence), they may be due to PCR errors rather than real editing events.

For AsCas12a, 179 independent events were recovered (84 events from tissue maintained at 27°C and 95 independent events from calli incubated at 37°C for a week). However, only two samples from heat-treated tissue and one from control temperature show editing: one of the heat-treated samples shows biallelic editing (one allele has a 7-bp deletion and the other presents a 4-bp deletion), and the second sample has a 10-bp deletion in one of the alleles. The sample maintained at 27°C shows minimal modifications, with 90% of the reads showing no editing and 10% of the reads have either a 5- (5.4%) or 8-bp deletion (4.6%). No editing was detected at any of the predicted off-target sites.

Finally, Mb3Cas12a sequencing shows nearly null editing rates when tissue was maintained at 27°C (2.96%), contrasting with the visual inspection score that showed editing rates of 8%. This difference can only be explained by phenotyping errors on the first replicate, where pale yellow calli were scored as white. When tissue was heat-treated, sequencing results are the same as with visual scoring (27.4% by sequencing vs 16% visually). No evidence of editing was detected at any of the off-target sites, except for one sample (PDS_Rep3_27C_Mb3_C8-2) from one replicate kept at 27°C, which shows a 45-bp deletion in 8.1% of the reads at the third off-target site.

## 4 Discussion

We evaluated the efficiency, specificity, and temperature effects of the most frequently used Cas variants—Cas9 and Cas12a (also named Cpf1)—used in plant science (Modrzejewski et al., 2019), by evaluating editing frequencies in PDS gene using rice embryogenic calli. The PDS gene has been used as a marker to infer CRISPR-Cas activity in several species (Li et al., 2013; Shan et al., 2014; Cai et al., 2015; Nishitani et al., 2016; Pan et al., 2016; Nakajima et al., 2017; Odipio et al., 2017; Kaur et al., 2018; Hooghvorst et al., 2019; Wilson et al., 2019; Bánfalvi et al., 2020; Wang et al., 2020; Lu and Tian, 2022), as PDS knockout mutants have an easily noticeable albino phenotype. Rice embryogenic tissue was the chosen system because, as a mass of pluripotent cells, calli have the ability to induce *de novo* shoot bud regeneration, being highly relevant to developmental biology. Protoplast, in contrast, can be isolated from virtually any plant tissues (Reed and Bargmann, 2021) and provide a valid system for rapid, high throughput *in vivo* studies of gene expression and evaluation of genome editing efficacy (Poddar et al., 2020), but its preparation is laborious, time-consuming, and there’s a lack of streamlined protocols to regenerate plants after protoplast transformation (Reed and Bargmann, 2021).

The sequence of the target-specific crRNA, its structural properties (Bortesi et al., 2016; Hassan et al., 2021; Riesenberg et al., 2022), and free energy changes on the target PAM context (Corsi et al., 2022) dictate the DNA target recognition mechanism of Cas protein and consequently have an impact on the Cas protein efficiency at on- and off-target sites. Accordingly, gene editing is facilitated by the availability of software (Stemmer et al., 2015; Chari et al., 2017; Liu et al., 2017; Kim et al., 2018; Listgarten et al., 2018; Labun et al., 2019; Zhu and Liang, 2019) that can identify different PAM sites, predict the editing efficiency at that site, and predict if off-target editing will take place. Finding unique target sites helps minimize the occurrence of such off-target edits, but truly unique target sites are difficult to find in highly duplicated genomes. The ability to choose from an assortment of PAM sites helps identify sites that are less likely to result in off-target effects. While it is unlikely that an off-target edit in a plant will lead to a novel risk (Graham et al., 2020), such edits remain a cause of public and regulatory concern, so they are best avoided.

Here we monitored the three best potential off-target sites for each target-specific sequence. In general, no edits were detected in any of the off-target sites selected for any of the different Cas nucleases tested. Only one single event (PDS_Rep3_27C_Mb3_C8-2) from one specific replicate kept at 27°C, had 8.1% of the reads edited at the third off-target site. Some samples had the same exact same SNP in a recurrent position, always far from the expected cleavage site. These results can be explained by Illumina sequencing interpretation errors. We observed a decrease in average quality of R2 reads as compared to R1 reads in Illumina MiSeq platform paired end sequencing. The degree of quality reduction varied between reads of the same library. The low quality of R2 reads reduces sequencing depth and thus confounds data interpretation. Similar findings have been reported previously (Schirmer et al., 2015; Chen et al., 2018; Ramakodi, 2021).

As mentioned above, we aimed to test the impact of temperature on Cas9/Cas12a activity, as these nucleases were isolated from different bacteria having an optimal growth range, from 35°C to 45°C (Angelos, 2010; Chang et al., 2010; Harnett et al., 2011; Hudson, 2014; Gera and McIver, 2013), and these temperatures are substantially higher than the 20°C–28°C temperature range that is best suited for plant tissue culture and transformation. In fact, when solely assessed visually, nuclease activity appears to be reduced at room temperature (22°C–28°C) (Moreno-Mateos et al., 2017; LeBlanc et al., 2018; Malzahn et al., 2019; Milner et al., 2020; Schindele and Puchta, 2020), with Cas12a being the most negatively impacted. Editing efficiency is reported to improve when transformed plant tissue is incubated at higher temperature for a shorter period of time (Jordan et al., 2021; Kurokawa et al., 2021). In our work, when all edits were considered (i.e., including monoallelic edits detected only after amplicon sequencing), the beneficial effect of elevated temperature only held true for Mb3Cas12a. Temperature did not alter the number of edits achieved in our samples with either SpCas9 or SaCas9; what differed was the ratio of monoallelic to biallelic edits in SaCas9. Likewise, in a recent study, Banakar et al. (2022) found no significant temperature dependency on the activity of 6 different CRISPR-Cas ribonucleoproteins (RNP), including SpCas9, AsCas12a, and LbCas12a.

The low editing efficiency of AsCas12a in our results is consistent with three previous reports (Hu et al., 2017; Kim et al., 2017; Banakar et al., 2020): two of the reports failed to detect any activity in rice T_0_ plants whereas the second one reported barely any AsCas12a-induced mutations in soybean protoplasts. In contrast, another study found AsCas12a has higher temperature sensitivity and lower nuclease activity than LbCas12a and FnCas12a under comparable conditions, but still achieved up to 93% editing frequency in T_0_ rice mutant lines when tissue was selected at 32°C (Malzahn et al., 2019).

Finally, despite having evaluated 289 events obtained with two different tracrRNAs – none of the events recovered after St1Cas9 transformation were edited. This apparent null activity in our results for St1Cas9 sharply contrasts with previous studies conducted in Arabidopsis at 22°C reporting successful gene editing with similar mutation frequencies as SpCas9 (Steinert et al., 2015; Steinert et al., 2017). At the same time, St1Cas9 has been used successfully for editing mammalian cells cultured at 37°C (Esvelt et al., 2013; Kleinstiver et al., 2015). Thus, we can only hypothesize that the use of a non-ideal specific target site sequence could have negatively impacted St1Cas9 activity.

Of all the endonucleases studied here, *S. pyogenes* Cas9 (SpCas9) is the most robust and widely used Cas9 endonuclease (Jaganathan et al., 2018; Chen et al., 2019; El-Mounadi et al., 2020; Nidhi et al., 2021) and is also the most efficient in our tests in terms of mono- and biallelic edits. It is relatively temperature insensitive. In contrast, the proportion of biallelic editing increased with temperature for SaCas9 from *Staphylococcus aureus.* When Mb3Cas12a from *M. bovoculi* was used, total editing increased with temperature, but proportionally more biallelic editing was obtained at 37°C. Collectively, these three endonucleases used at the proper temperature provide a measure of flexibility when the number of suitable PAM sites is limited, as can happen in complex plant genomes. At the same time, the widely different reports on the efficiency of any given endonuclease shows that a lot of the parameters for their efficient use are still not understood.

## Data Availability

The datasets presented in this study can be found in online repositories. The names of the repository/repositories and accession number(s) can be found below: https://www.ncbi.nlm.nih.gov/bioproject, PRJNA892053.

## Supplementary material

Supplementary Figure S1 Identification of protospacer sequences targeting the rice phytoene desaturase (OsPDS) gene using different Cas9/Cas12a variants. Schematic representation of the rice PDS gene with the mRNA and CDS sequences indicated as blue and yellow block arrows, respectively. Each triangle represents a protospacer sequence. Each specific target site sequence is colored according to their activity (on-target) or specificity (off-target) score. The annotation for the activity score is a gradient from red to green with lower values in red and higher values green. The change of coloring for the off-target score ranges from red (indicating lower specificity) to green (high specificity or fewer off-target sites). Black indicates that the algorithm available in Geneious (https://www.geneious.com) cannot predict the activity scores for that specific Cas variant. Selected guide RNA sequences are highlighted by pink color boxes.

Supplementary Table S1 Relevant sequences used in this study.

Supplementary Table S2 List of oligonucleotides used in this study.

Supplementary Table S3 Molecular verification of editing from all newly formed hygromycin-resistant events.
