# Supplementary figures and images for: Editing efficiencies with Cas9 orthologs, Cas12a endonucleases, and temperature in rice
Source: Front Genome Ed. 2023 Mar 17;5:1074641. doi: 10.3389/fgeed.2023.1074641 (PMC10080323; doi:10.3389/fgeed.2023.1074641)

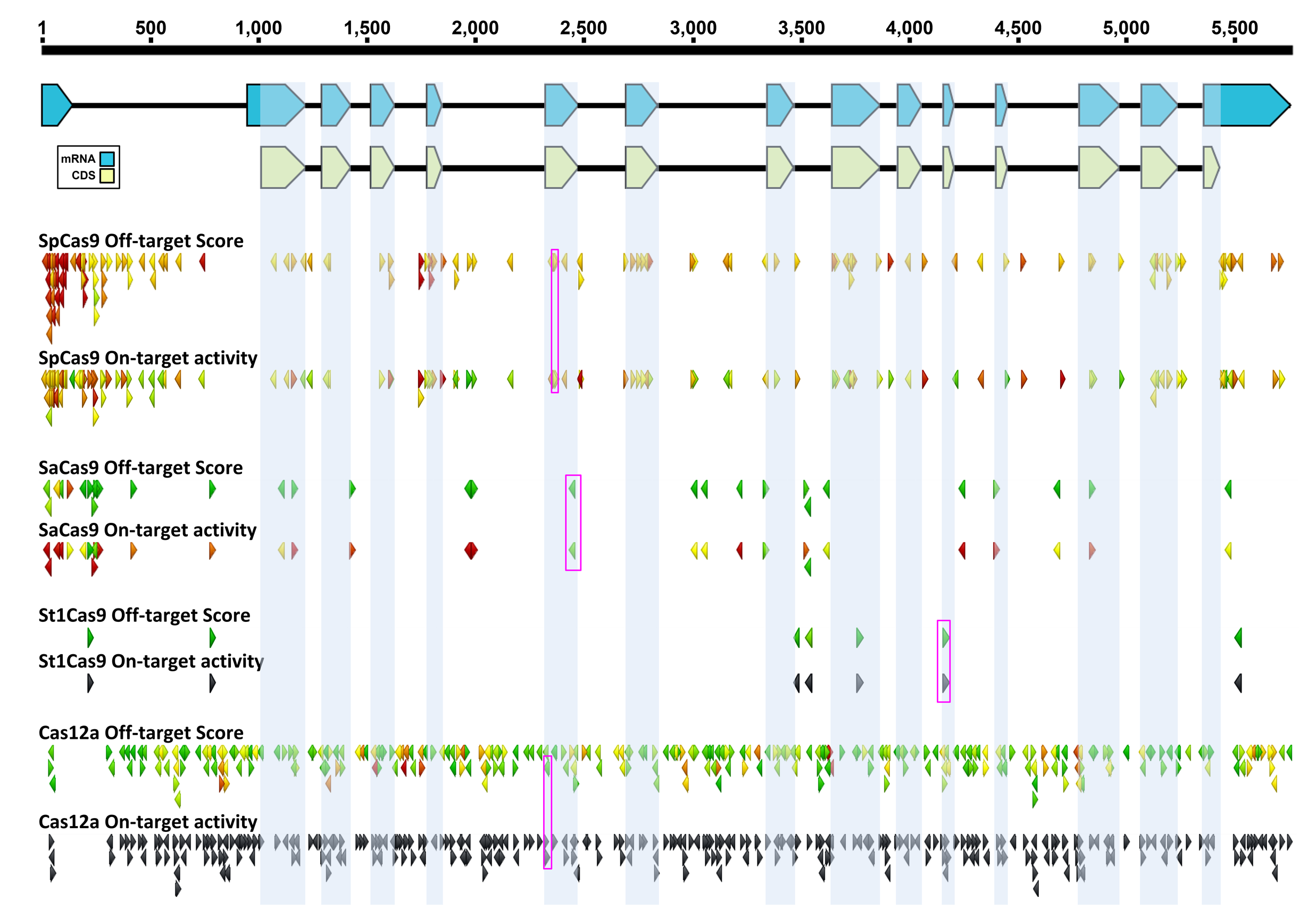

Supplement: Supplementary file 3 [file Image1.TIF]
